# Impact Monitoring for Aircraft Smart Composite Skins Based on a Lightweight Sensor Network and Characteristic Digital Sequences

**DOI:** 10.3390/s18072218

**Published:** 2018-07-10

**Authors:** Lei Qiu, Xiaolei Deng, Shenfang Yuan, YongAn Huang, Yuanqiang Ren

**Affiliations:** 1 Research Center of Structural Health Monitoring and Prognosis, State Key Lab of Mechanics and Control of Mechanical Structures, Nanjing University of Aeronautics and Astronautics, Nanjing 210016, China; lei.qiu@nuaa.edu.cn (L.Q.); dengxl@nuaa.edu.cn (X.D.); renyuanqiang@nuaa.edu.cn (Y.R.); 2 State Key Lab of Digital Manufacturing Equipment and Technology, School of Mechanical Science and Engineering, Huazhong University of Science and Technology, Wuhan 430074, China; yahuang@hust.edu.cn

**Keywords:** aircraft smart skin, composite structure, impact monitoring, lightweight sensor network, characteristic digital sequence

## Abstract

Due to the growing use of composite materials in aircraft structures, Aircraft Smart Composite Skins (ASCSs) which have the capability of impact monitoring for large-scale composite structures need to be developed. However, the impact of an aircraft composite structure is a random transient event that needs to be monitored on-line continuously. Therefore, the sensor network of an ASCS and the corresponding impact monitoring system which needs to be installed on the aircraft as an on-board device must meet the requirements of light weight, low power consumption and high reliability. To achieve this point, an Impact Region Monitor (IRM) based on piezoelectric sensors and guided wave has been proposed and developed. It converts the impact response signals output from piezoelectric sensors into Characteristic Digital Sequences (CDSs), and then uses a simple but efficient impact region localization algorithm to achieve impact monitoring with light weight and low power consumption. However, due to the large number of sensors of ASCS, the realization of lightweight sensor network is still a key problem to realize an applicable ASCS for on-line and continuous impact monitoring. In this paper, three kinds of lightweight piezoelectric sensor networks including continuous series sensor network, continuous parallel sensor network and continuous heterogeneous sensor network are proposed. They can greatly reduce the lead wires of the piezoelectric sensors of ASCS and they can also greatly reduce the monitoring channels of the IRM. Furthermore, the impact region localization methods, which are based on the CDSs and the lightweight sensor networks, are proposed as well so that the lightweight sensor networks can be applied to on-line and continuous impact monitoring of ASCS with a large number of piezoelectric sensors. The lightweight piezoelectric sensor networks and the corresponding impact region localization methods are validated on the composite wing box of an unmanned aerial vehicle. The accuracy rate of impact region localization is higher than 92%.

## 1. Introduction

Aircraft smart skin is considered to be an important technology to improve the flight and safety performance of future advanced aircrafts [1,2,3,4,5]. The basic principle of aircraft smart skin is to integrate a large number of sensors, actuators and microprocessors with the aircraft skin structure so that the flight environment, operational conditions, health status and other information of the aircraft structure can be monitored and the corresponding performance of the aircraft can be controlled adaptively. This can realize self-diagnosis, self-learning, self-healing and other capabilities of the aircraft structures. Structural Health Monitoring (SHM) is one of the key capabilities of an aircraft smart skin [3,4,5].

In recent two decades, composite materials have been widely applied to aircraft structures, especially to the structures of large aircrafts due to their high specific stiffness and strong resistance to fatigue and corrosion as well as their design flexibility [6]. Furthermore, the scale of composite structures of large aircrafts is often large such as the wing, fuselage and tail etc. The latest Airbus A350XWB, taken as an example, is composed of 53% composites [7], and the Boeing B787 aircraft uses 50% of composite materials [8]. However, for aircraft composite structures, the impact of external objects such as bird strikes, hail, gravel and maintenance tools can easily lead to internal delamination, matrix cracking and even fiber fracture [9,10]. These kinds of damage can cause a significant reduction of the mechanical properties and load-bearing capacity of composite structures, and will further pose a potential threat to the structural safety. Therefore, the development of the ASCS which has the SHM capability to monitor impacts and damage is needed to ensure the safety and reduce the maintenance cost of large aircraft composite structures [3,4].

Since the impact of an aircraft composite structure is a random transient event, it needs to be monitored instantaneously when it happens. Therefore, the sensor network of ASCS which is applied to impact monitoring must be lightweight and reliable to realize on-line and continuous impact monitoring. In addition, the corresponding impact monitoring system needs to be connected to the sensor network and it also needs to be installed on the aircraft as an on-board device for online and continuous impact monitoring. Therefore, it must also meet the requirements of light weight, low power consumption and high reliability [11]. Among the existing SHM methods, the piezoelectric (referred to as PZT) sensors and guided wave based impact monitoring method is a popular one [12,13,14,15,16,17,18,19,20,21,22] because PZT sensors have a large monitoring range by constructing a monitoring network and can work in a passive way for sensing impact response signals of guided wave on a monitored structure without any power source. However, it is still difficult for a PZT sensor network and the corresponding impact monitoring system to meet the requirements mentioned above at the same time because of the following main problems:

(1) The PZT sensor network of ASCSs often contains a large number of PZT sensors. Firstly, each PZT sensor needs at least one lead wire to transmit impact response signals. Therefore, the PZT sensor network has a large number of lead wires, which introduce large additional weight to the aircraft. Secondly, each PZT sensor often needs to occupy at least one monitoring channel of an impact monitoring system. A large number of PZT sensors require an impact monitoring system which has a large number of monitoring channels. This results in a large size, high power consumption and high complexity of the impact monitoring system leads. For example, when the PZT sensor network of ASCS consists of 100 PZT sensors, there are at least 100 signal transmitting lead wires and the impact monitoring system has 100 monitoring channels. This makes the performance and cost of the sensor network and the impact monitoring system difficult to be accepted for on-line and continuous impact monitoring.
(2) Most of the impact monitoring methods [12,13,14,15,16,17,18,19,20,21,22] aim at high-precision impact localization such as geometry-based localization, energy-based localization and impact imaging-based localization. These methods need to obtain complete impact response signals from PZT sensors. To achieve this, a monitoring channel of an impact monitoring system often contains signal conditioning circuits and a signal acquisition circuit. When the PZT sensor network of an ASCS contains a large number of PZT sensors, the number of monitoring channels becomes very large. Furthermore, a complex computation module is also needed to process impact monitoring algorithms [23,24,25]. These factors increase the weight and power consumption of the impact monitoring system and they limit the application of ASCSs to on-line and continuous impact monitoring. This is the reason why these systems are mainly used in laboratory or ground applications [11,26].

To address problem (2) mentioned above, Yuan and Qiu et al. proposed a new miniaturized Impact Region Monitor (IRM) based on Characteristic Digital Sequences (CDSs) [27,28,29,30]. The principle of IRM is to convert the impact response signals output from PZT sensors into CDSs directly through a simple array of comparators. Then the IRM uses the impact region localization algorithm which is based on the CDSs to achieve impact region monitoring. The key feature of the IRM is that it uses comparators to replace the signal conditioning circuits, and uses a Field Programmable Gate Array (FPGA) to replace the signal acquisition circuits and computation module of a traditional impact monitoring system. Thus it greatly reduces the size and power consumption of the traditional impact monitoring system. However, problem (1) mentioned above still remains unsolved regarding the application of the IRM to ASCS.

Considering the methods to address problem (1), the continuous sensor concept proposed by Kirikera and Schulz et al. [31,32] is a feasible approach. In the continuous sensor, PZT sensors are adopted to monitor acoustic emission signals to realize acoustic source localization. The PZT sensors in the same row and column are respectively connected in series and constitute a single continuous sensor. Multiple continuous sensors form a continuous sensor network. A traditional acoustic emission system is used to acquire and process the signals output from the continuous sensor network. The continuous sensor greatly reduces the number of lead wires of the PZT sensor network and it also greatly reduces the number of channels of the acoustic emission system, but the acoustic emission system still has large size and high power consumption, which causes that the continuous sensor cannot be applied to on-line and continuous impact monitoring.

Based on the discussion above, this paper combines the concept of the continuous sensor with the IRM and CDSs. The lightweight sensor network is proposed which has three network architectures including continuous series sensor network, continuous parallel sensor network and continuous heterogeneous sensor network. The methods to realize impact region localization, which are based on the CDSs and the different architectures of the lightweight sensor network, are proposed as well so that the lightweight sensor network can be connected to the IRM for on-line and continuous impact monitoring of ASCS with a large number of PZT sensors. Finally, the lightweight sensor network and the impact region localization methods are validated on the composite wing box of an Unmanned Aerial Vehicle (UAV) and show high accuracy rate of impact region localization.

In this paper, the principle of impact region monitoring of the ASCS based on the IRM and CDSs are briefly introduced first in Section 2. Then in Section 3, three kinds of lightweight sensor networks are proposed. After that, the impact region localization methods based on the three kinds of lightweight sensor networks and CDSs are proposed and evaluated on a glass fiber laminate plate in Section 4. In Section 5, the validation on the UAV composite wing box is performed and discussed. Finally, the conclusions are presented in Section 6.

## 2. The Principle of Impact Region Monitoring of the ASCS

### 2.1. The Architecture of the IRM

**Figure 1:**
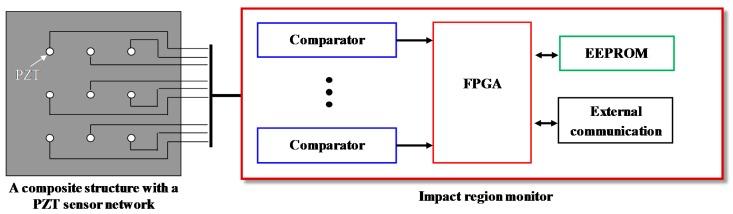
The architecture of the IRM.

The simplified architecture of the IRM [27,28,29] is given in Figure 1. The PZT sensors are connected to a voltage comparator array. When an impact happens on the composite structure, the output of the PZT sensor network can be converted to the CDSs by using the voltage comparator array with a threshold voltage. Then a FPGA is used to acquire the CDSs and perform the impact region localization. The impact monitoring results are stored in an Electrically Erasable Programmable Read-Only Memory (EEPROM). The SHM users can use the external communication such as RS232 to download the impact monitoring results from the IRM whenever they want.

As mentioned before, due to the simplicity of the hardware and software, the IRM has small size and low power consumption. Thus it can be applied to ASCS for on-line and continuous impact monitoring.

### 2.2. The Principle of Impact Region Localization

**Figure 2:**
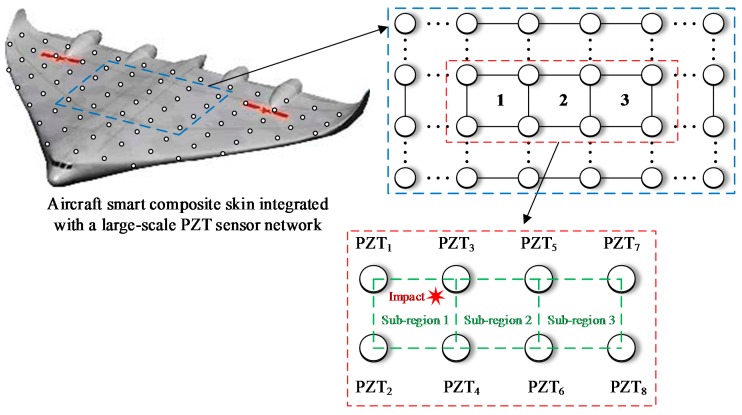
The schematic diagram of an ASCS integrated with a large-scale PZT sensor network.

**Figure 3:**
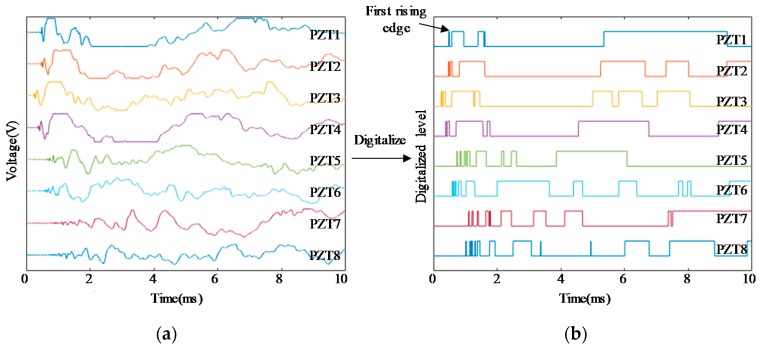
An example of impact response signals and the corresponding CDSs: (**a**) Impact response signals and (**b**) CDSs.

The schematic diagram of an ASCS which is integrated with a large-scale PZT sensor network is shown in Figure 2. To illustrate the principle of impact region monitoring, a small region which contains the eight PZT sensors numbered from PZT_1_ to PZT_8_ is selected to be an example. The eight PZT sensors form three impact monitoring sub-regions. In a traditional impact monitoring system, when an impact happens at the sub-region surrounded by PZT_1_, PZT_2_, PZT_3_ and PZT_4_, the impact response signals of the eight PZT sensors are acquired as shown in Figure 3a and then impact localization methods can be applied to get the accurate position of the impact in the sub-region. For the IRM, there is no need to get the complete impact response signals but to get the CDSs as shown in Figure 3b. It can be noted that some features of the CDSs can be used to locate the impact sub-region. For example, according to the order of the arrival time of the first rising edge appearing in each CDS, the first four PZT sensors (PZT_1_, PZT_2_, PZT_3_ and PZT_4_) can be recognized so that the impact can be localized in the actual impact sub-region surrounded by the four PZT sensors.

**Figure 4:**
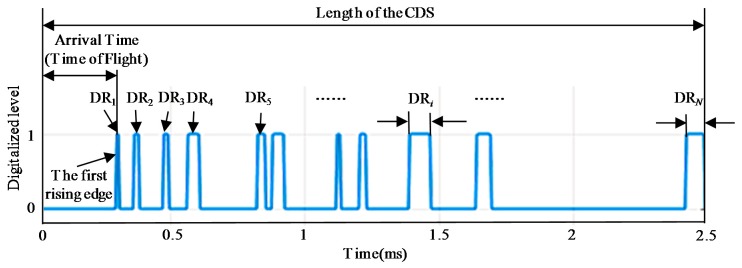
Illustration of the features DR and OFRE of a CDS.

To increase the accuracy rate of impact region localization and support a large impact monitoring network, the algorithm based on energy-weighted region localization [33] can be adopted. In the algorithm, the Energy-Weighted Factor (EWF) given by Equation (1) is defined to characterize the extent to which the sensor is affected by an impact. It is obtained based on two features extracted from a CDS, namely the Duration of the Rise (DR) and the Order of the First Rising Edge (OFRE). As shown in Figure 4 and Equation (2), DR is the total duration time of the high digital level ‘1’ of the CDS and represents the impact energy. The first rising edge appears when the digitalized level of a CDS changes from 0 to 1 for the first time. Arrival time (time of flight) refers to the duration from 0 to the time at which the first rising edge appears. OFRE describes the order of the arrival time of the first rising edge among all the PZT sensors. Due to the complexity of the aircraft composite structures and the environmental noise, the two features are combined to calculate EWF for a more reliable estimation of the impact sub-region. Obviously, EWF values of the four PZT sensors around the impact sub-region should be the maximal ones among all the PZT sensors. Thus the impact influence on every sub-region can be evaluated by summing the EWF values of the PZT sensors. The sub-region which has the highest EWF summing value is the impact sub-region:

(1) $$\mathrm{EWF}=\frac{\mathrm{DR}}{\mathrm{OFRE}}$$

(2) $$\mathrm{DR}=\sum_{i=1}^{N}{\mathrm{DR}}_{i}$$

where DR*_i_* is the duration of the *i*-th high digital level ‘1’ of a CDS.

## 3. Lightweight Sensor Network

As mentioned before, the PZT sensor network shown in Figure 2 will have a large number of lead wires and occupy a large number of impact monitoring channels. The additional weight of the network to the aircraft and the power consumption and size of the IRM will be difficult to be accepted for real applications. Considering this point, the concept of a continuous sensor network can be used. If some of the PZT sensors shown in Figure 2 can be connected to be a continuous sensor, the number of lead wires and occupied impact monitoring channels will be greatly reduced. However, even if it can be done, the PZT sensor network still needs to monitor the impact region accurately based on the CDSs. Therefore, the signal features of the continuous sensor are studied by an experiment first in this section. Then the three kinds of lightweight sensor networks are proposed and discussed.

### 3.1. Signal Features of Continuous Sensor

**Figure 5:**
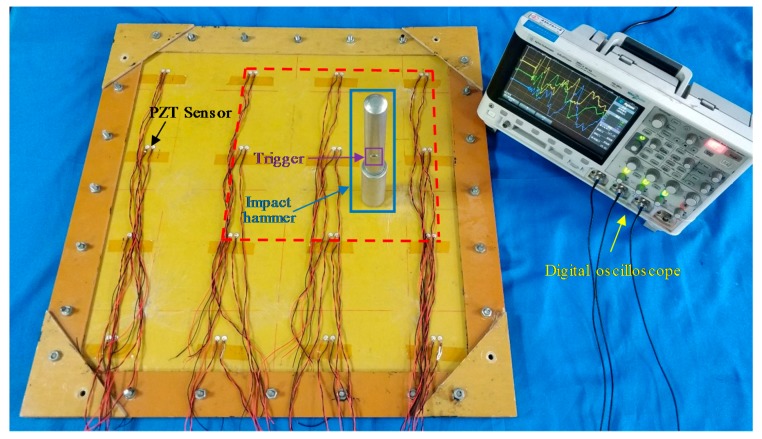
The experimental setup for studying the features of the continuous sensor.

To study the signal features of the continuous sensor, an experiment is conducted on a glass fiber laminate plate, which is shown in Figure 5. There are 32 PZT sensors on this plate and only nine sensors in the red dashed box are used in this experiment. The material of the PZT sensor is PZT-5A. The diameter and thickness of the PZT sensor are 8 mm and 0.48 mm, respectively. An impact device shown in Figure 5 is adopted to apply impact to the plate. The head of the impact device pops out to apply an impact when the trigger is pulled. The impact hammer has three impact energy degrees which can produce impact energy of 0.21, 0.62 and 1.31 J approximately to the impacted surface. An MSOX 3014A digital oscilloscope (Agilent, Santa Clara, CA, United States) is adopted to acquire the complete impact response signals of the sensors directly.

**Figure 6:**
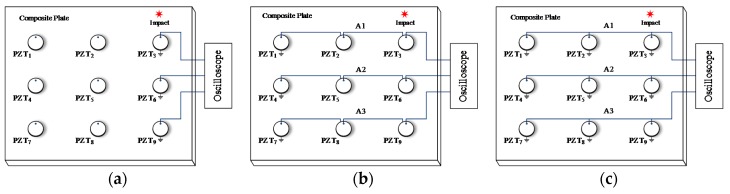
Different sensor connection types: (**a**) Independent connection; (**b**) Series connection; (**c**) Parallel connection.

The PZT sensors on the composite plate are connected to the digital oscilloscope in three different sensor connection types as shown in Figure 6a–c respectively. In Figure 6a, three PZT sensors are connected to the digital oscilloscope independently. In Figure 6b, the three PZT sensors in the same row are connected in series to be a continuous series sensor and connected to one acquisition channel of the digital oscilloscope. The three continuous series sensors are numbered from A1 to A3. In Figure 6c, the three PZT sensors in the same row are connected in parallel to be a continuous parallel sensor and connected to one acquisition channel of the digital oscilloscope. The three continuous parallel sensors are also numbered from A1 to A3. The sampling rate of the digital oscilloscope is set to be 1 MHz.

**Figure 7:**
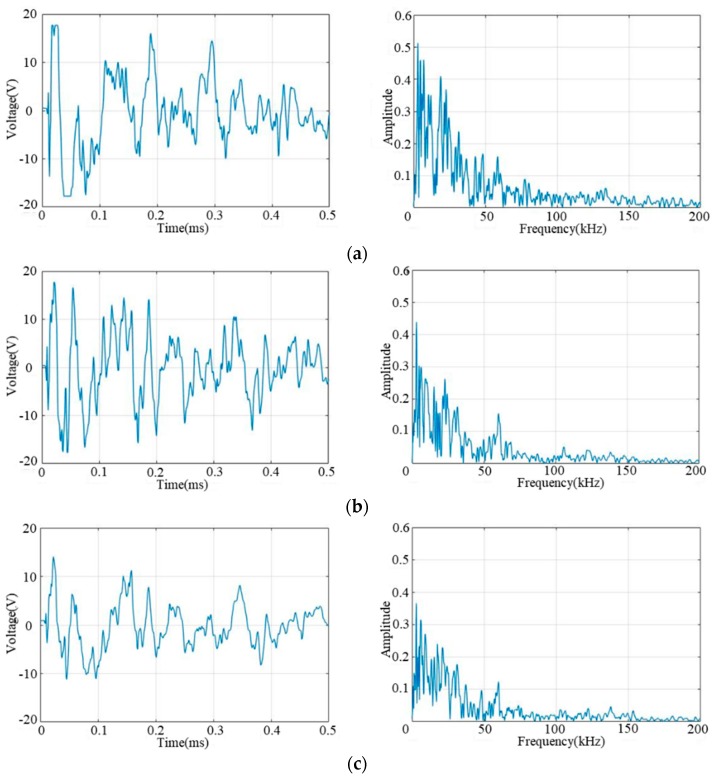
Typical impact response signals and frequency spectrums of different sensor connection types: (**a**) The impact response signal and frequency spectrum of PZT_3_ in independent connection type; (**b**) The impact response signal and frequency spectrum of A1 in series connection type; (**c**) The impact response signal and frequency spectrum of A1 in parallel connection type.

For each sensor connection type, an impact with the same energy is applied to the same position on the composite plate. The typical impact response signal and the corresponding frequency spectrum of the three sensor connection types are shown in Figure 7. It can be seen from the frequency spectrum that the bandwidth of the impact response signal of different sensor connection types is nearly the same, which indicates that the continuous sensor keeps the main frequency feature of the impact response signal. It can be also seen that the maximum amplitude of the signal is gradually decreasing from the independent sensor to the continuous series sensor and the continuous parallel sensor. However, based on the principle of impact region monitoring, the arrival time of the signal is the key feature. Thus the amplitude changes of the impact response signal introduce little influence on the impact region localization.

**Figure 8:**
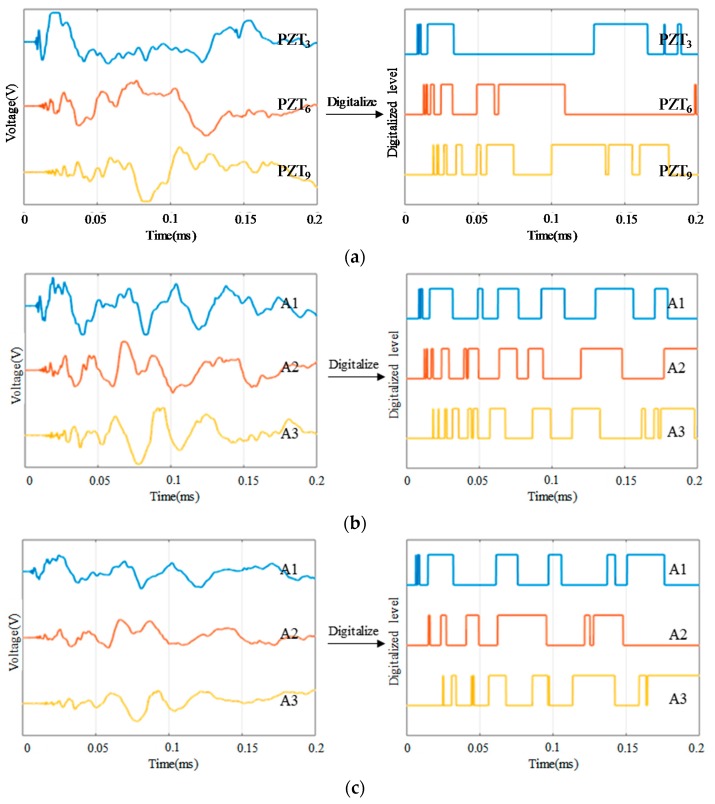
Typical impact response signals and CDSs of different sensor connection types under impact energy level 1: (**a**) The impact response signals and CDSs of PZT_3_, PZT_6_ and PZT_9_ in independent connection type; (**b**) The impact response signals and CDSs of A1, A2 and A3 in series connection type; (**c**) The impact response signals and CDSs of A1, A2 and A3 in parallel connection type.

**Figure 9:**
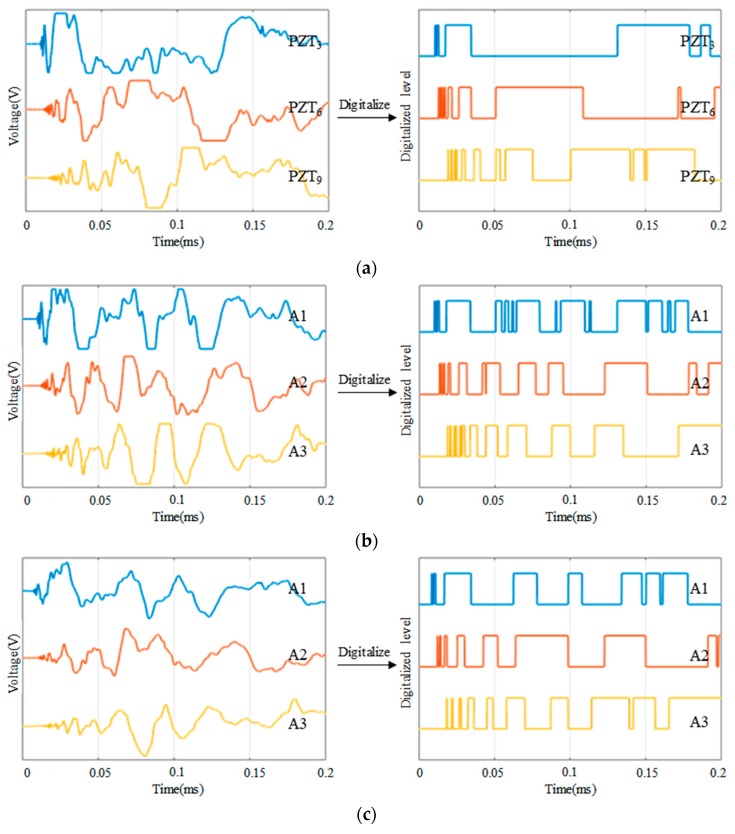
Typical impact response signals and CDSs of different sensor connection types under impact energy level 2: (**a**) The impact response signals and CDSs of PZT_3_, PZT_6_ and PZT_9_ in independent connection type; (**b**) The impact response signals and CDSs of A1, A2 and A3 in series connection type; (**c**) The impact response signals and CDSs of A1, A2 and A3 in parallel connection type.

**Figure 10:**
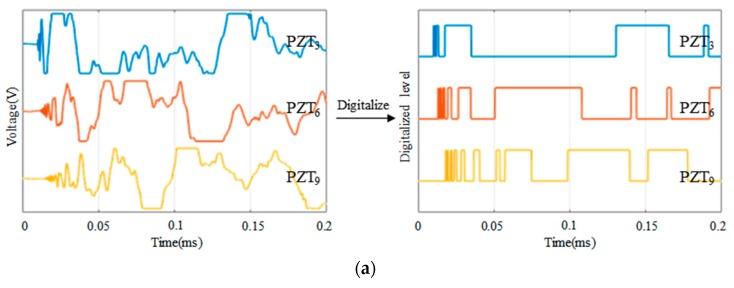

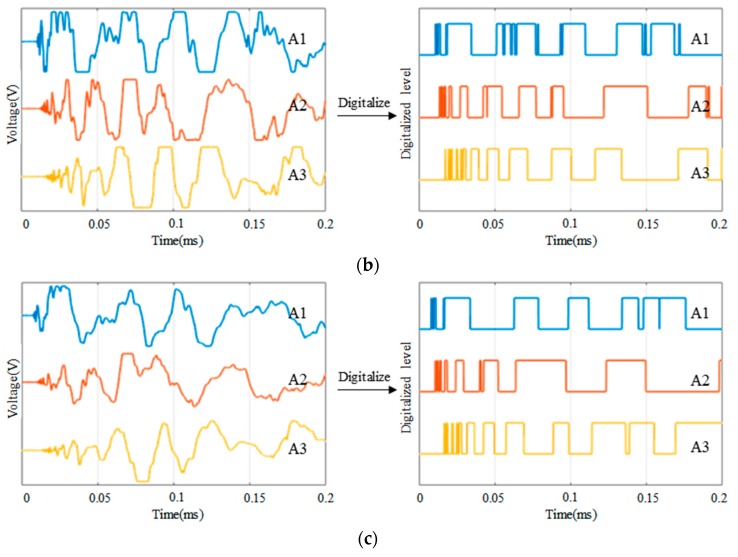
Typical impact response signals and CDSs of different sensor connection types under impact energy level 3: (**a**) The impact response signals and CDSs of PZT_3_, PZT_6_ and PZT_9_ in independent connection type; (**b**) The impact response signals and CDSs of A1, A2 and A3 in series connection type; (**c**) The impact response signals and CDSs of A1, A2 and A3 in parallel connection type.

Figure 8, Figure 9 and Figure 10 compare the arrival time of the impact response signals of different sensor connection types under different impact energy levels. As it can be seen from the voltage response of these signals that the arrival time of the impact response signals of the continuous sensors is corresponding to the actual impact position and is correct. By using a software threshold voltage of 3 V, the voltage responses of these signals are converted to the CDSs. It can be seen that the OFRE of the continuous sensors is correct as well. Based on these results, it indicates that connecting PZT sensors in series or in parallel will not change the arrival time and the OFREs of the impact response signals and CDSs. Therefore, the principle of impact region monitoring based on the CDSs can be applied to the continuous sensors.

Furthermore, it can be seen from the comparison of these figures under different impact energy levels that the voltage of impact response signals will increase with the impact energy levels getting higher. Therefore, the DRs of the CDSs will also increase due to the increasing energy levels. However, the arrival time and OFREs will not change when the impact energy level increases. Considering that the impact energy level 1 has a low energy of 0.21 J and the features of CDSs under this level are in accord with expectations, ordinary impact under higher energy levels can be monitored by using the abovementioned impact region localization algorithm.

In addition, the capacitance values of the continuous sensors with different number of PZT sensors are measured using a 79-III Digital Multimeter (FLUKE, Everett, WA, United States). The results are given in Table 1. As it can be seen that the continuous sensor connection will change the capacitance of the whole PZT sensor network. For the parallel connection type, the capacitance is increased. Furthermore, the impedance of the PZT sensor network is reduced due to the parallel connection of PZT sensors. It will reduce the impact response sensitivity when a large number of PZT sensors are parallel connected. This point can also be known by comparing Figure 7c with Figure 7a. For the series connection type, the capacitance is reduced but the impact response sensitivity is still reduced due to the change of impedance. However, the impact response sensitivity of the series connection type is still higher than that of the parallel connection type. Based on this point, the series connection of PZT sensors is better.

### 3.2. The Architecture of the Lightweight Sensor Network

**Figure 11:**
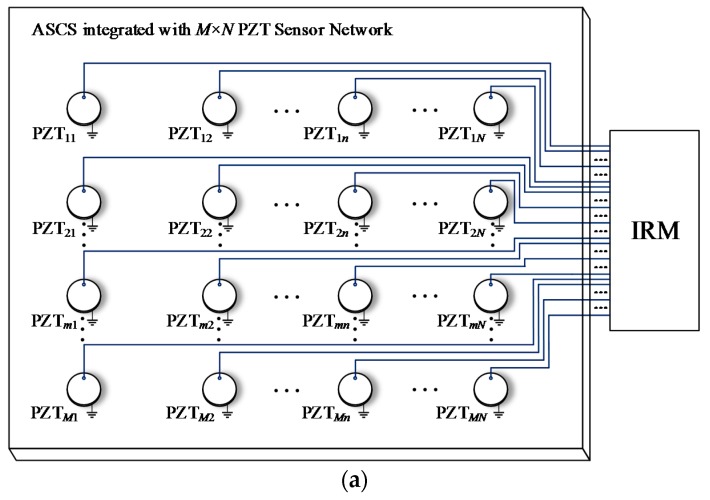

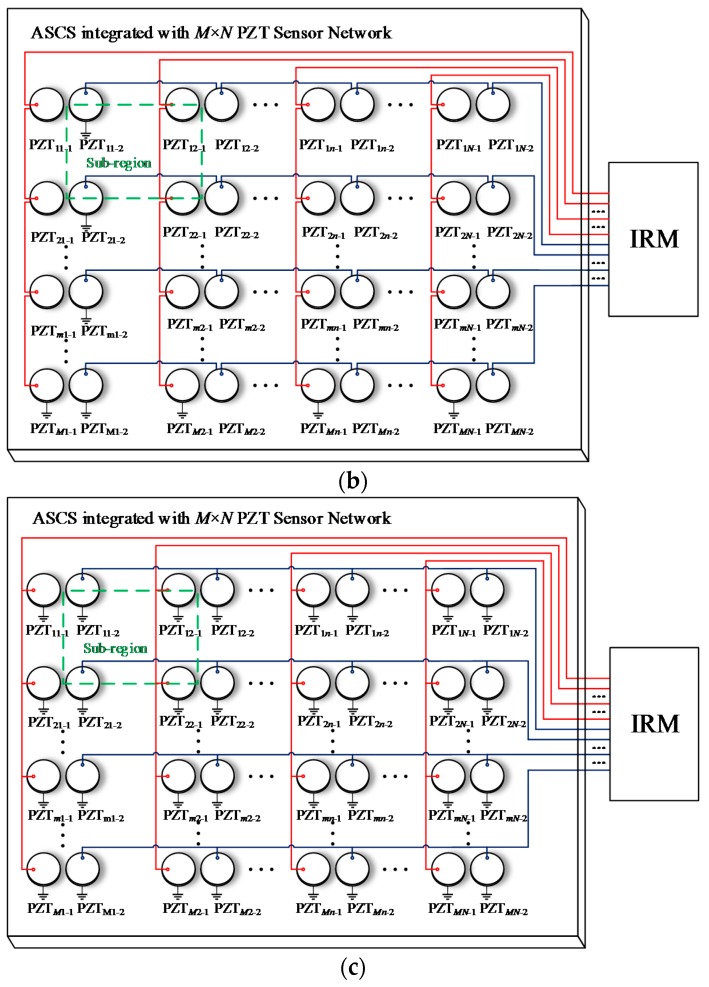
The architecture of: (**a**) Normal PZT sensor network; (**b**) CSSN; (**c**) CPSN.

Figure 11a presents a Normal Sensor Network (NSN) for impact monitoring which is placed on a composite structure and is connected to the IRM. The NSN contains *M* × *N* PZT sensors. Each PZT sensor uses one lead wire to transmit impact response signal and occupies one monitoring channel of the IRM. As mentioned before, when a large number of PZT sensors are used, the PZT sensor network will have a large number of lead wires and occupy a large number of monitoring channels correspondingly. To address this problem, three kinds of lightweight sensor networks are proposed including Continuous Series Sensor Network (CSSN), Continuous Parallel Sensor Network (CPSN) and Continuous Heterogeneous Sensor Network (CHSN).

The architecture of CSSN shown in Figure 11b is constructed by *M* × *N* PZT sensor pairs. A pair of PZT sensors numbered as PZT*_mn_*_-1_ and PZT*_mn_*_-2_ are placed close to each other instead of a single PZT sensor of the NSN. The PZT sensors numbered as PZT*_mn_*_-1_ on the left side of a PZT sensor pair in the same column are connected in series with each other. The PZT sensors numbered as PZT*_mn_*_-2_ on the right side of a PZT sensor pair in the same row are connected in series with each other as well. Each four adjacent sensor pairs construct an impact monitoring sub-region. It can be seen that the CSSN contains 2 × *M* × *N* PZT sensors and there are *M* + *N* lead wires and the network occupies *M* + *N* monitoring channels of the IRM.

The architecture of CPSN which is constructed by *M* × *N* PZT sensor pairs is shown in Figure 11c. It is close to the CSSN. The only difference is that the corresponding sensors are connected in parallel but not in series. The number of PZT sensors and occupied monitoring channels is the same as those of the CSSN.

It is clear that the CSSN and CPSN can greatly reduce the number of lead wires and the occupied monitoring channels, which is meaningful for reducing the weight and complexity of the ASCS and the IRM. However, these two kinds of lightweight sensor networks need to use twice as many sensors as the NSN. Meanwhile, they need to place the lead wires in both horizontal and vertical direction on a composite structure. For a real aircraft composite structure, stiffeners are often used which make it hard to integrate the two networks with the structure. Taking a composite wing as an example, when the two networks are required to be placed on the inner surface of the wing skin to avoid the influence of airflow in flight, they need to go through the stiffeners.

**Figure 12:**
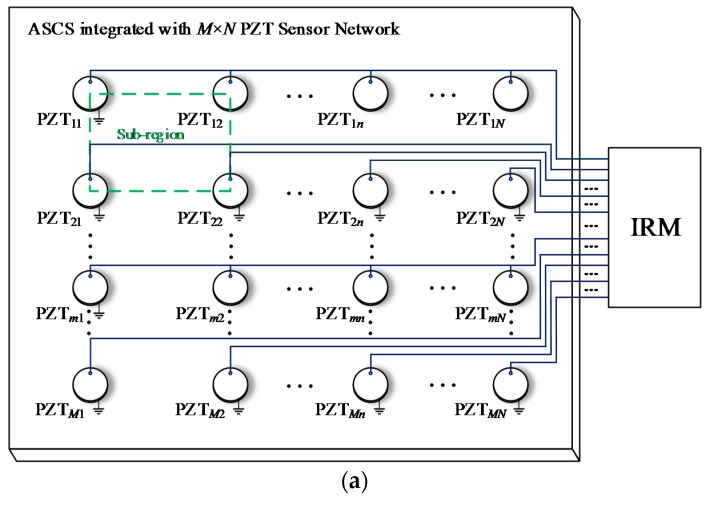

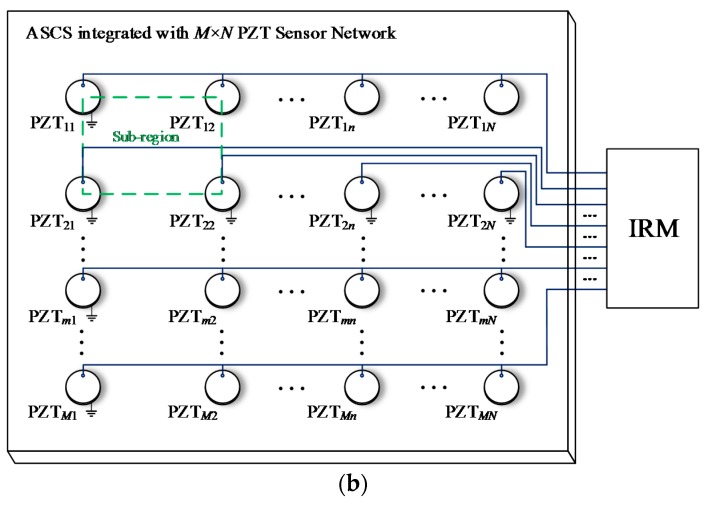
The architecture of CHSN when: (**a**) *N* is even; (**b**) *N* is odd.

To avoid the abovementioned problems, CHSN is proposed as shown in Figure 12, which is constructed by *M* × *N* PZT sensors. The PZT sensors at the odd rows are connected in series to form a continuous sensor, the PZT sensors at even rows remain independent. When *N* is even, there are (*M* × *N* + *N*)/2 lead wires and (*M* × *N* + *N*)/2 occupied monitoring channels. When *N* is odd, there are (*M* × *N* − *M* + *N* + 1)/2 lead wires and (*M* × *N* − *M* + *N* + 1)/2 occupied monitoring channels. Each four adjacent sensors construct an impact monitoring sub-region. It can be noted that CHSN only has lead wires in one direction, which is helpful to the network placement of the ASCS.

Table 2 compares the three lightweight sensor networks with the NSN in different network sizes (*M* ≥ *N* ≥ 2). Compared to the NSN, the three lightweight sensor networks can greatly reduce the lead wires and occupied monitoring channels and the CHSN can achieve this point without increasing the number of PZT sensors.

## 4. Impact Region Localization Method Based on Lightweight Sensor Network

### 4.1. Impact Region Localization Method

Based on the energy-weighted region location algorithm discussed in Section 2.2, two impact region localization methods based on CSSN/CPSN and CHSN, respectively, are proposed as follows.

**Figure 13:**
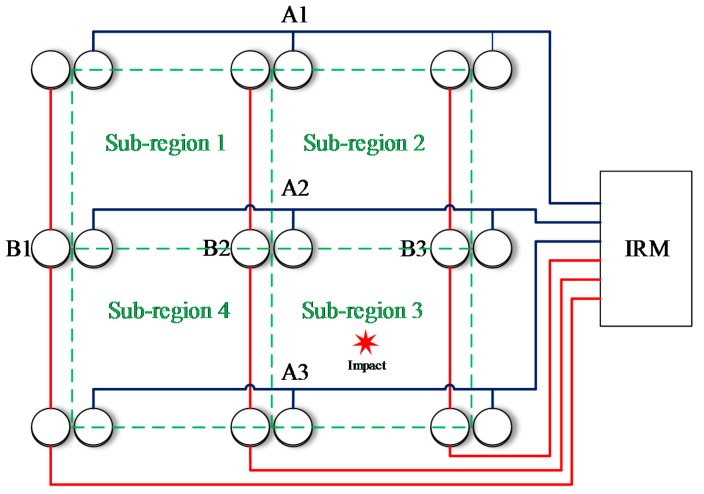
Schematic diagram of the sensor network for illustrating the impact region localization method based on the CSSN and CPSN.

**Figure 14:**
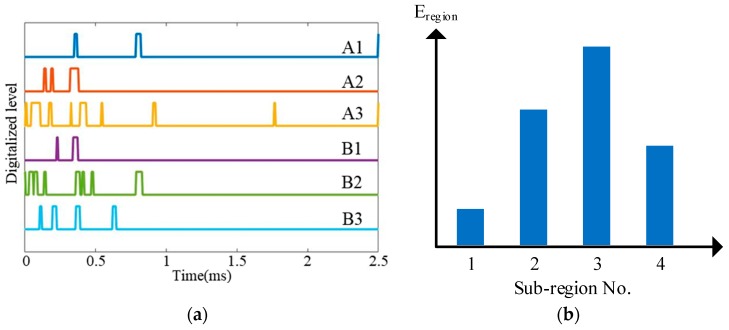
Impact region localization results based on the CSSN and CPSN: (**a**) CDSs and (**b**) E_region_ of sub-regions.

To illustrate the impact region localization method based on the CSSN/CPSN, a size of 3 × 3 lightweight sensor network is adopted to be an example as shown in Figure 13. There are 18 PZT sensors and they form six continuous series/parallel sensors numbered from A1 to A3 and B1 to B3 respectively. The continuous sensors are connected to the IRM. Four impact monitoring sub-regions can be constructed. A1, A2, B1 and B2 form the sub-region 1. A1, A2, B2 and B3 form the sub-region 2 and so on. When an impact happens on the structure, the IRM obtained six CDSs as shown in Figure 14a which correspond to the six continuous sensors. The features DR and OFRE of each CDS are calculated and the corresponding EWF is obtained based on Equation (1). The parameter E_region_ of an impact monitoring sub-region is defined to represent the sum of EWFs of the four continuous sensors which construct this sub-region. The results of E_region_ are given in Figure 14b. The sub-region which has the highest E_region_ is determined to be the impact sub-region.

**Figure 15:**
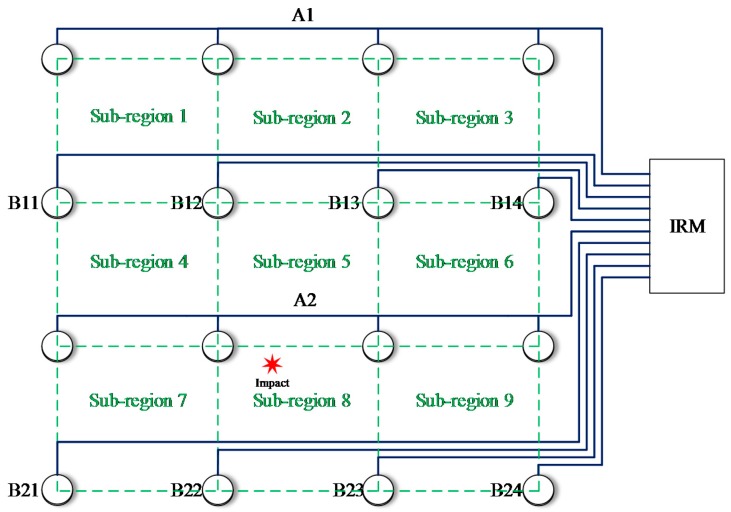
Schematic diagram of the sensor network for illustrating the impact region localization method based on the CHSN.

**Figure 16:**
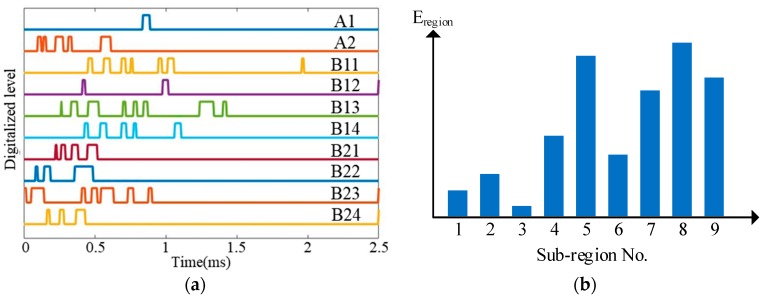
Impact region localization results based on the CHSN: (**a**) CDSs and (**b**) E_region_ of sub-regions.

To illustrate the impact region localization method based on the CHSN, a 4 × 4 size lightweight sensor network is adopted to be an example as shown in Figure 15. There are 16 PZT sensors. Among them, eight PZT sensors are used to form two continuous series sensors numbered as A1 and A2, and the other eight PZT sensors are used independently and they are numbered from B11 to B24. The two continuous sensors and all the independent PZT sensors are connected to the IRM. Nine impact monitoring sub-regions can be constructed. A1, B11 and B12 constitute the sub-region 1. A1, B12 and B13 constitute the sub-region 2 and so on. When an impact happens on the structure, the IRM obtains ten CDSs as shown in Figure 16a. The features DR and OFRE of each CDS are calculated and the corresponding EWF is obtained. The parameter E_region_ of the nine sub-regions are obtained and shown in Figure 16b. The sub-region which has the highest E_region_ is determined to be the impact sub-region.

### 4.2. Experimental Evaluation

**Figure 17:**
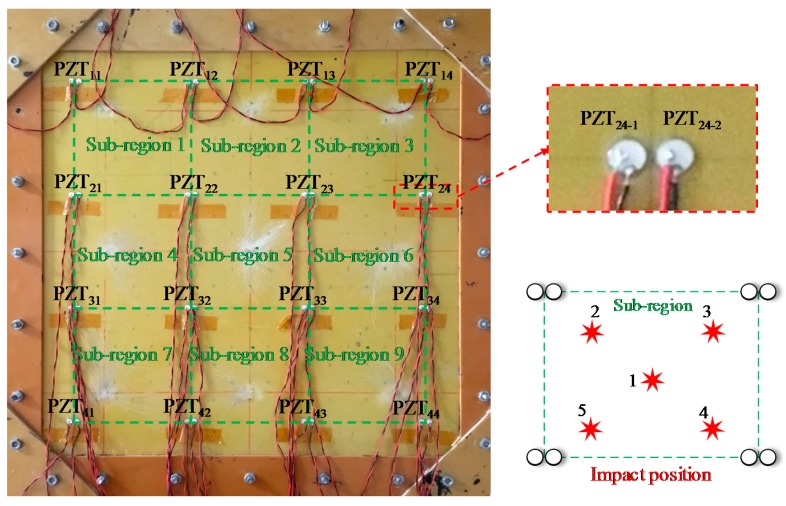
The glass fiber laminate plate and PZT sensors on it.

**Figure 18:**
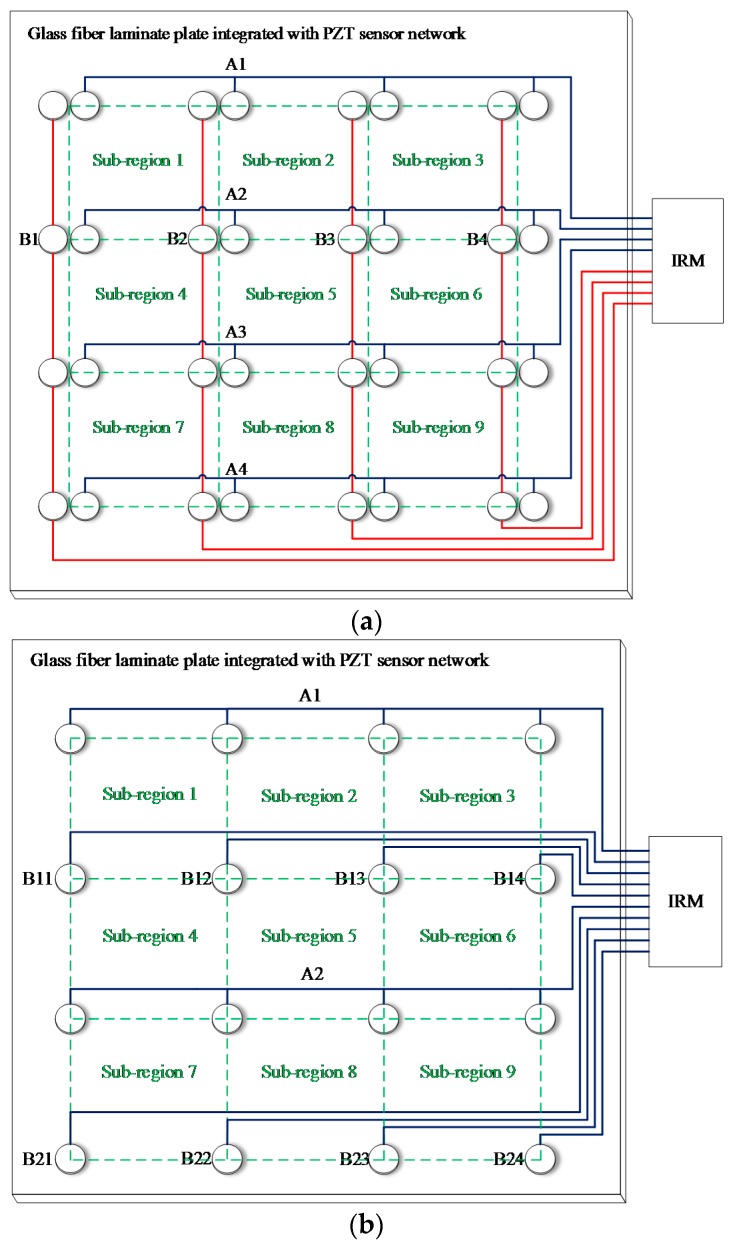
Schematic diagram of lightweight sensor networks placed on the glass fiber laminate plate: (**a**) CSSN/CPSN and (**b**) CHSN.

To evaluate the impact region localization methods based on CSSN/CPSN and CHSN, an experiment is performed on a 600 mm × 600 mm glass fiber laminate plate as shown in Figure 17. The thickness is 2 mm. Sixteen PZT sensor pairs including 32 PZT sensors are placed on the plate and they form nine monitoring sub-regions. In the experiment, the PZT sensors are used to construct the CSSN, CPSN and CHSN, respectively. The impact hammer shown in Figure 5 is also adopted to apply three different impact energy levels. On each lightweight sensor network, 225 impacts of one energy level are applied to the plate in total. In each sub-region, five impacts are applied to each position shown in Figure 17. Figure 18a is the schematic diagram of the sensor connection of the CSSN and CPSN. The 32 PZT sensors form eight continuous sensors numbered from A1 to A4 and B1 to B4. Figure 18b is the schematic diagram of the sensor connection of CHSN. It should be noted that only the PZT sensor on the right in a sensor pair is used. The PZT sensors in the first row and third row are used to form two continuous series sensors numbered as A1 and A2. The PZT sensors in the second row and fourth row are used independently and they are numbered from B11 to B24.

**Figure 19:**
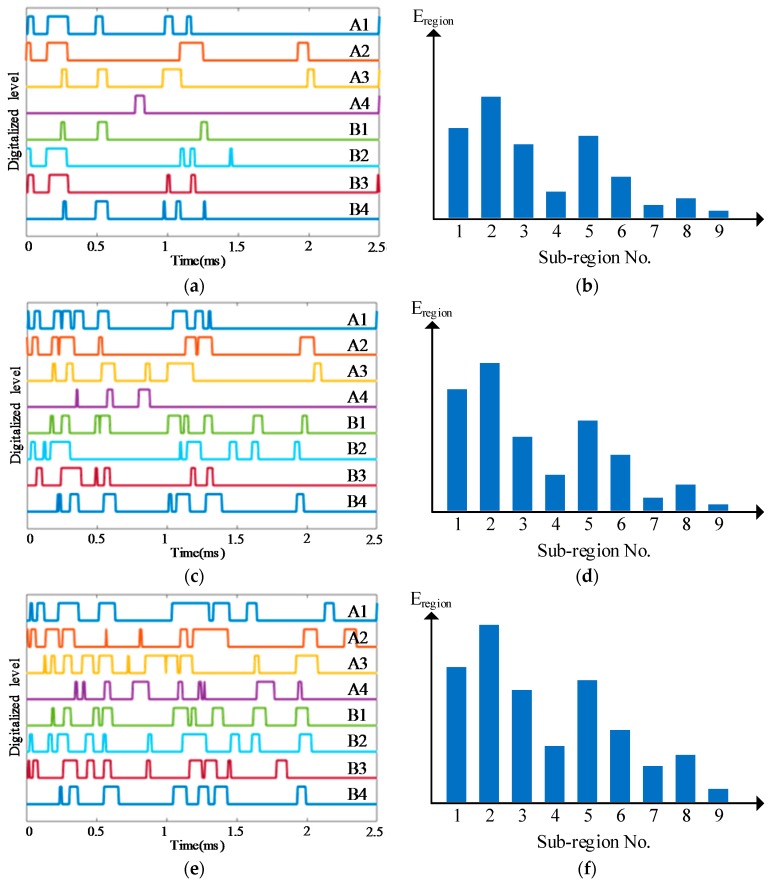
Typical impact region monitoring results on the glass fiber laminate plate based on CSSN under different energy levels: (**a**) CDSs and (**b**) E_region_ of all sub-regions under energy level 1; (**c**) CDSs and (**d**) E_region_ of all sub-regions under energy level 2; (**e**) CDSs and (**f**) E_region_ of all sub-regions under energy level 3.

**Figure 20:**
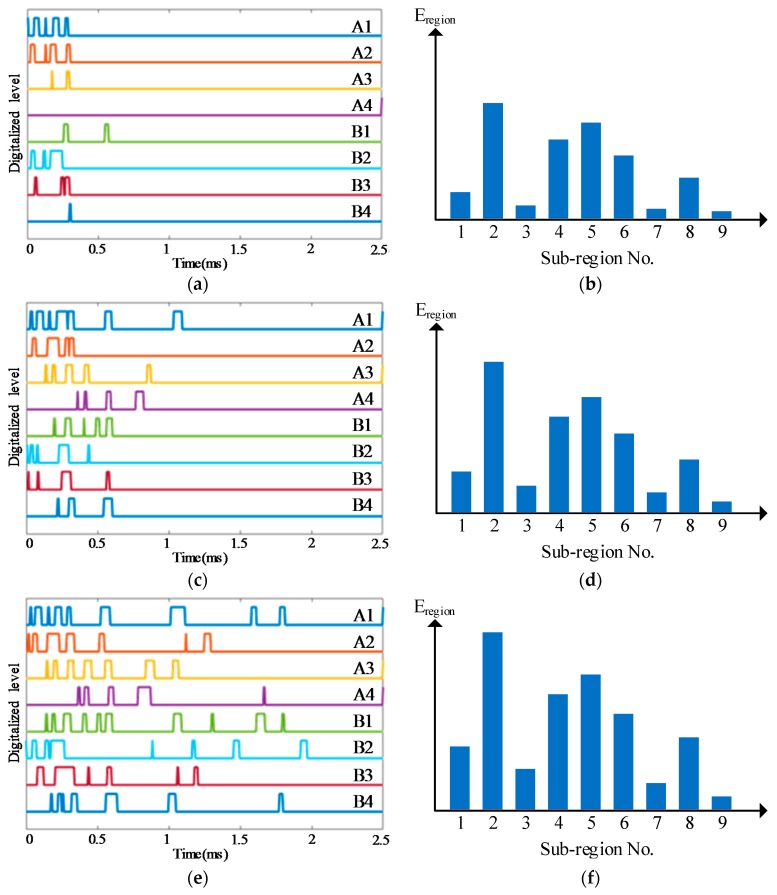
Typical impact region monitoring results on the glass fiber laminate plate based on CPSN under different energy levels: (**a**) CDSs and (**b**) E_region_ of all sub-regions under energy level 1; (**c**) CDSs and (**d**) E_region_ of all sub-regions under energy level 2; (**e**) CDSs and (**f**) E_region_ of all sub-regions under energy level 3.

**Figure 21:**
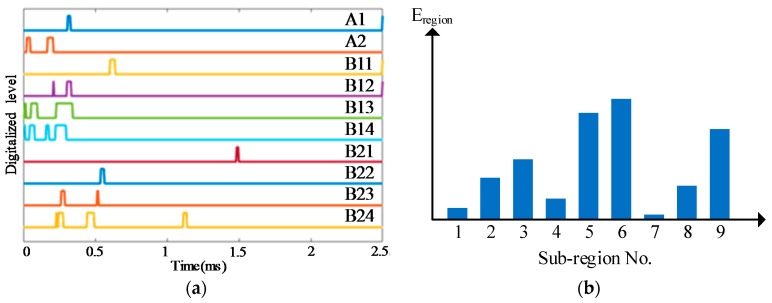

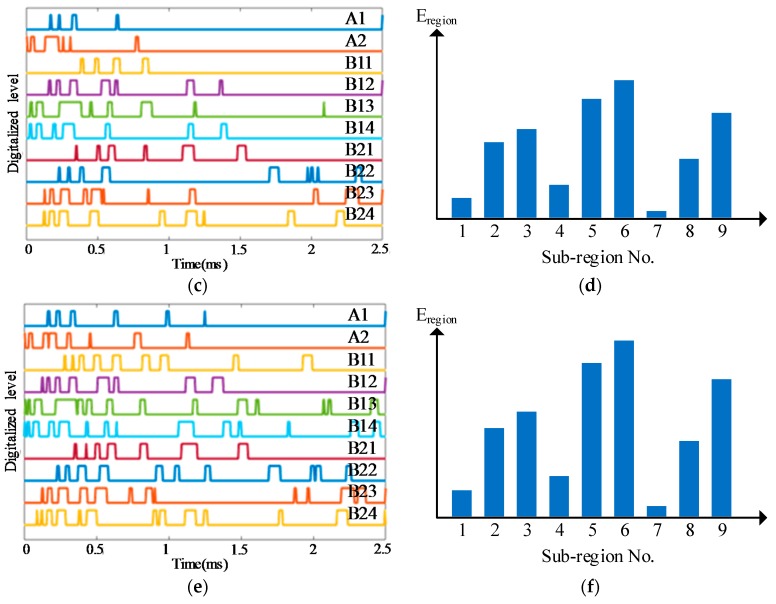
Typical impact region monitoring results on the glass fiber laminate plate based on CHSN under different energy levels: (**a**) CDSs and (**b**) E_region_ of all sub-regions under energy level 1; (**c**) CDSs and (**d**) E_region_ of all sub-regions under energy level 2; (**e**) CDSs and (**f**) E_region_ of all sub-regions under energy level 3.

Some typical impact region monitoring results of the three kinds of lightweight sensor networks under different impact energy levels are given in Figure 19, Figure 20 and Figure 21. The results indicate that the CDSs are correctly obtained and the corresponding E_reigon_ can represent the actual impact sub-region correctly as well.

Table 3 compares the number of impacts applied in the nine monitoring sub-regions and the number of impacts located correctly according to the impact region localization method under different impact energy levels. The results show that three kinds of lightweight sensor networks are feasible to locate impact sub-region with high accuracy under different impact energy levels, while impact of low energy may affect the accuracy rate. Considering that the impact energy of a real aircraft structure is often higher than that of this experiment, the impact accuracy rate is acceptable. In general, CHSN has the highest accuracy rate in three kinds of networks.

## 5. Validation on an UAV Composite Wing Box

**Figure 22:**
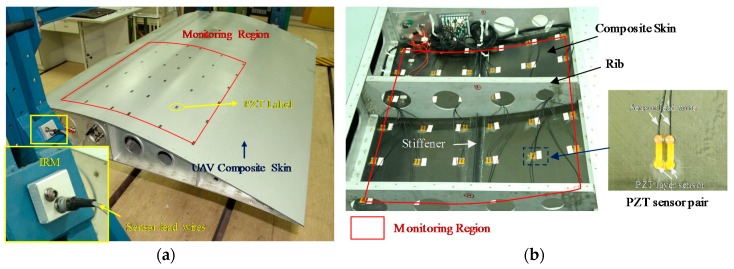
The UAV composite wing box, IRM and sensor placement: (**a**) The UAV composite wing box and the IRM and (**b**) Sensor placement.

To validate the lightweight sensor networks and the impact region localization methods on a real aircraft structure, an UAV composite wing box is adopted as shown in Figure 22. The lengths of the wing span and wing chord are 2.0 m and 1.5 m, respectively. The skin of the wing box is made of carbon fiber composite laminate and the thickness is 2.5 mm. 36 PZT sensor pairs including 72 PZT layer sensors [34] are placed on the inner surface of the composite skin.

**Figure 23:**
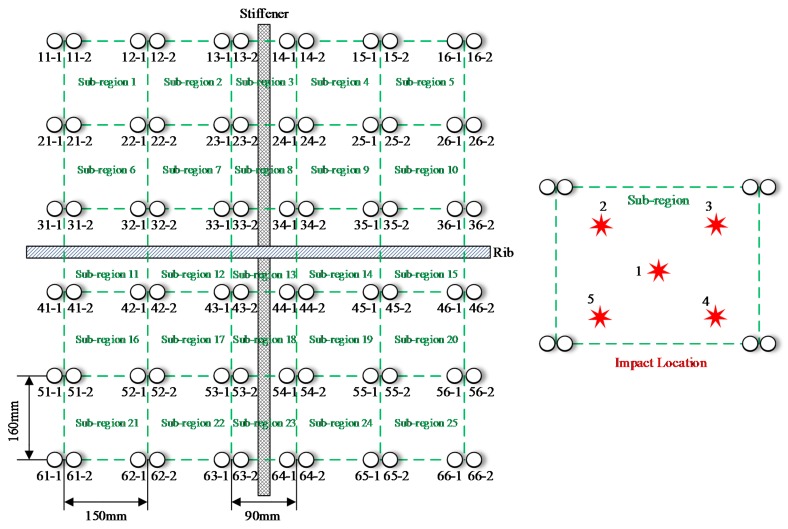
Illustration of PZT sensor placement on the UAV composite skin.

As shown in Figure 23, the area of the impact monitoring region covered by the PZT sensors is around 800 mm × 690 mm and 25 impact monitoring sub-regions are constructed by the PZT sensors. The PZT sensors are used to construct the CSSN, CPSN and CHSN respectively. The impact hammer shown in Figure 5 is also adopted to apply three different energy levels of impact. To each lightweight sensor network, 625 times of impact are applied to the composite skin under one energy level. In each sub-region, five impacts are applied to each position shown in Figure 23.

**Figure 24:**
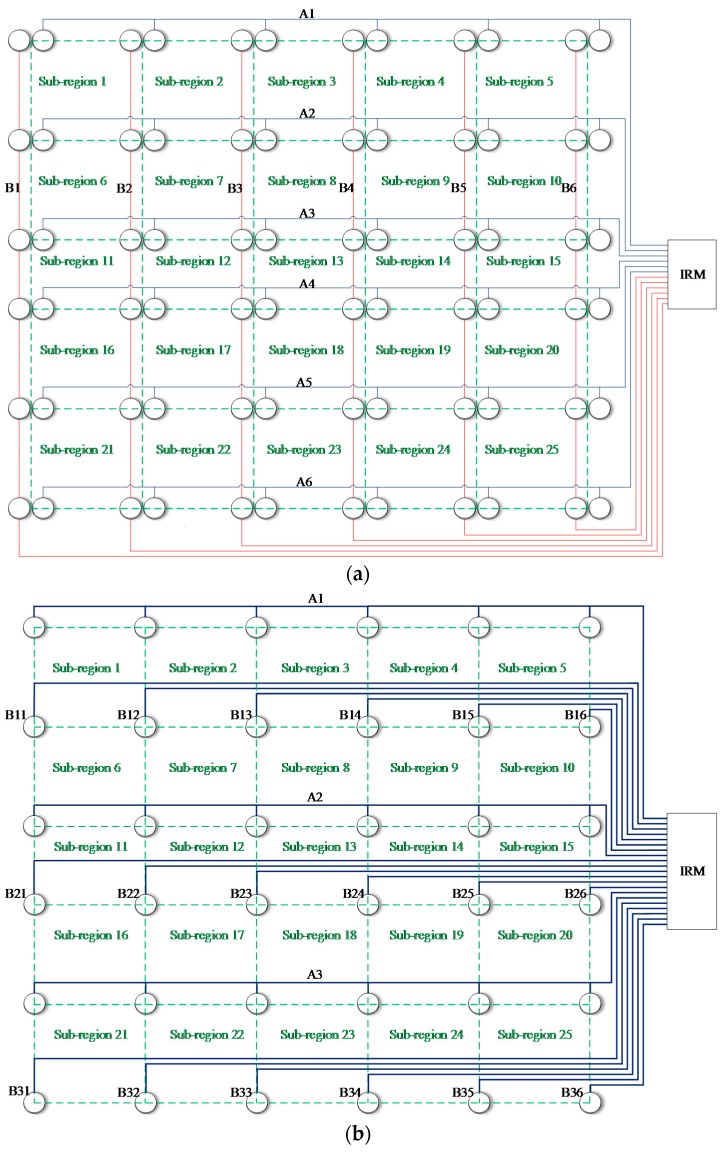
Schematic diagram of the lightweight sensor networks placed on the UAV composite skin: (**a**) CSSN/CPSN and (**b**) CHSN.

Figure 24a is the schematic diagram of the sensor connection of the CSSN and CPSN. The 72 PZT layer sensors construct 12 continuous sensors numbered from A1 to A6 and B1 to B6. Figure 24b is the schematic diagram of the sensor connection of the CHSN. The PZT sensor on the right in a PZT sensor pair is used.

The three lightweight sensor networks are connected to the IRM, respectively. For the CSSN and CPSN, 12 channels of the IRM are used. For the CHSN, 21 channels of the IRM are used. The voltage threshold of the IRM used in this validation is 3 V.

**Figure 25:**
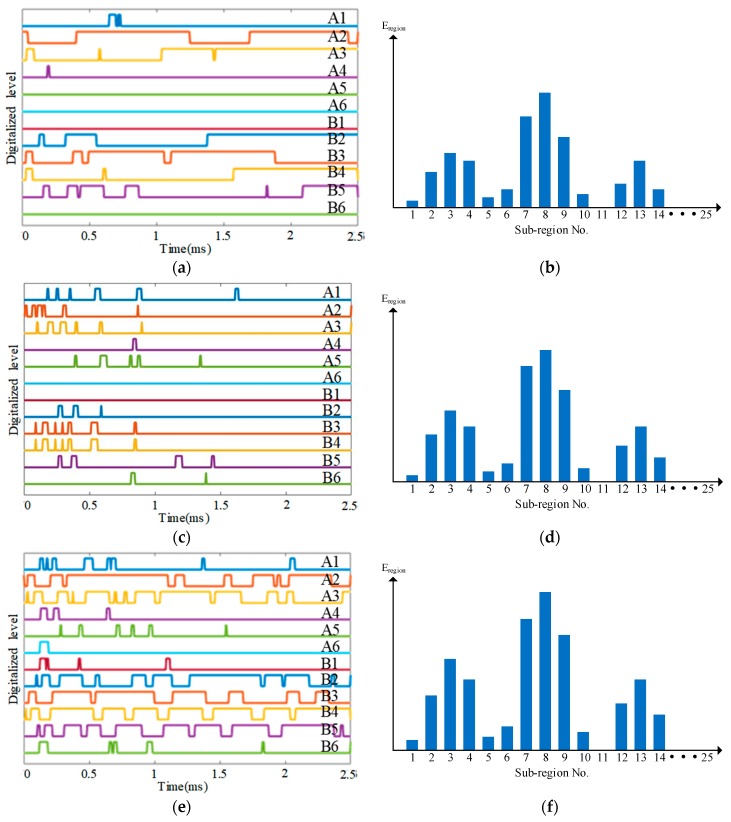
Typical impact region monitoring results on the UAV composite skin based on CSSN under different energy levels: (**a**) CDSs and (**b**) E_region_ of all sub-regions under energy level 1; (**c**) CDSs and (**d**) E_region_ of all sub-regions under energy level 2; (**e**) CDSs and (**f**) E_region_ of all sub-regions under energy level 3.

**Figure 26:**
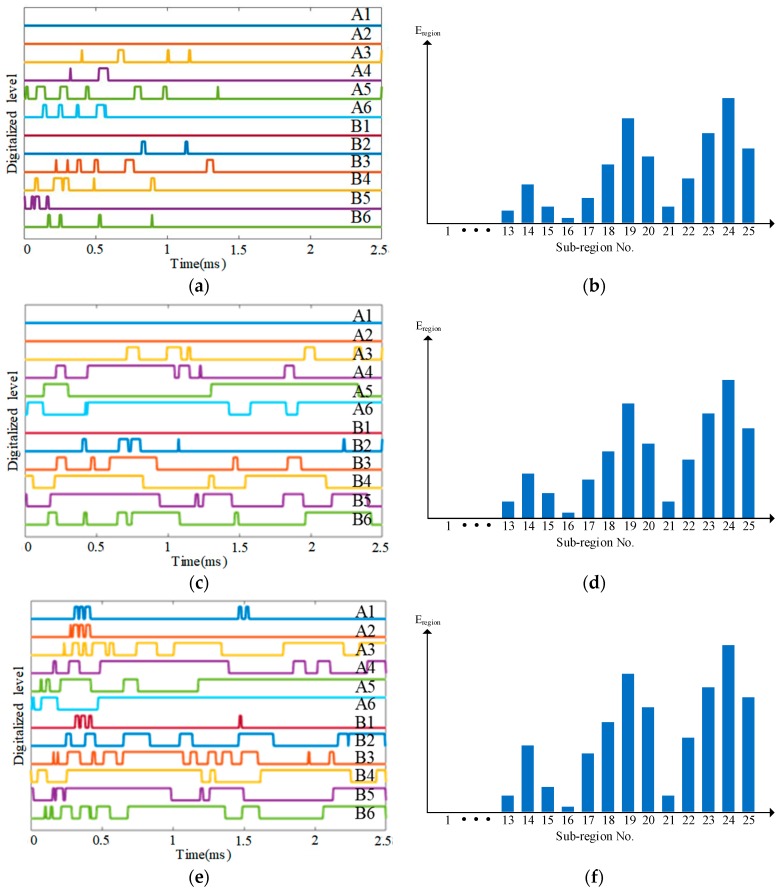
Typical impact region monitoring results on the UAV composite skin based on CPSN under different energy levels: (**a**) CDSs and (**b**) E_region_ of all sub-regions under energy level 1; (**c**) CDSs and (**d**) E_region_ of all sub-regions under energy level 2; (**e**) CDSs and (**f**) E_region_ of all sub-regions under energy level 3.

**Figure 27:**
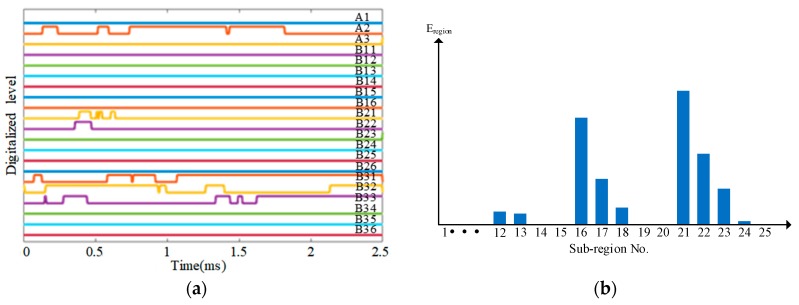

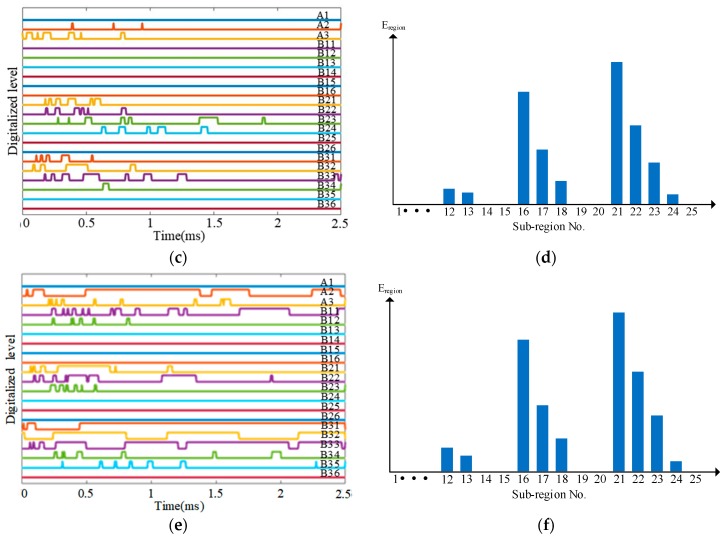
Typical impact region monitoring results on the UAV composite skin based on CHSN under different energy levels: (**a**) CDSs and (**b**) E_region_ of all sub-regions under energy level 1; (**c**) CDSs and (**d**) E_region_ of all sub-regions under energy level 2; (**e**) CDSs and (**f**) E_region_ of all sub-regions under energy level 3.

Some typical impact region monitoring results of the three lightweight sensor networks under different energy levels are given in Figure 25, Figure 26 and Figure 27. It can be seen that some CDSs are zero because the distance between the corresponding PZT layer sensors and the impact is too long so that the maximum voltage of the impact response signals is lower than the voltage threshold of the IRM. Overall speaking, the results indicate that the CDSs are correctly obtained and the corresponding E_reigon_ can represent the actual impact sub-region correctly as well.

Table 4 shows the number of impacts applied in the 25 monitoring sub-regions and the number of impacts located correctly according to the impact region localization method. In general, the localization accuracy rate of the three lightweight sensor networks is higher than 92% under all impact energy levels. In addition, the impact localization accuracy rates of different impact energy levels are nearly the same. The results show that the lightweight sensor networks and the corresponding impact region localization methods are feasible to be applied to aircraft structures with high monitoring accuracy rate.

## 6. Conclusions

This paper proposes three kinds of lightweight sensor networks including the CSSN, CPSN and CHSN for the lightweight realization of the ASCS for impact monitoring. The basic principle of the lightweight sensor network is to connect some of the PZT sensors of the network in series or in parallel so that these PZT sensors can share the same lead wire to transmit impact response signals. Therefore, the three kinds of lightweight sensor networks can greatly reduce the lead wires of the PZT sensor network and the occupied monitoring channels of the IRM, thus reducing the additional weight of the ASCS to an aircraft and the complexity and power consumption of the IRM. Particularly, the CHSN can achieve this point without increasing the number of PZT sensors.

The key feature of lightweight sensor network to monitor impacts is to convert the output of the lightweight sensor network to the CDSs by the IRM. Correspondingly, the impact region localization methods based on the CDSs and different architectures of the lightweight sensor network are proposed. With this, the lightweight sensor network can be finally connected to the IRM for on-line and continuous impact monitoring of the ASCS integrated with a large number of PZT sensors. A real UAV composite wing box is adopted to validate the performance of the methods and the validation results show that the impact region can be localized with high accuracy rate which is higher than 92%. Though the principle of the lightweight sensor network and impact region localization methods of the ASCS are proposed and the capability is validated on a real aircraft composite structure in this paper, the research is still at the preliminary stage. The work given as follows is planned to be performed in the near future:

(1) Compared with the independent sensor connection, the continuous sensor connection will change the capacitance and impedance of the whole PZT sensor network. This effect may reduce the monitoring accuracy and efficiency of the lightweight sensor networks so that the scalability of the lightweight sensor networks is reduced. This point will be theoretically analyzed and experimentally studied in the ongoing work.
(2) The accuracy of impact region localization methods on more real complex aircraft structures and under more complex noise environment will be further studied.
(3) A large-scale and flexible lightweight sensor network will be developed based on the principle of flexible and stretchable sensors [34,35,36] and the method of how to integrate the large-scale lightweight sensor network with the aircraft composite skin will be studied so as to realize a real lightweight ASCS.
(4) The IRM will be further improved so that it can be realized flexibly and can be integrated with the lightweight sensor network to be an integrated and lightweight impact monitoring network system.
(5) Furthermore, the three kinds of lightweight sensor networks proposed by this paper are sparse PZT sensor networks. They can also be applicable to some strategies of active monitoring based on dense PZT sensor networks [22,37,38,39,40,41]. Therefore, more different types of PZT sensor network and the corresponding active monitoring strategies will be studied.

## Figures and Tables

**Table 1:** Capacitance of PZT_3_, PZT_6_ and PZT_9_ in independent connection type, series connection type and parallel connection type.

Connection Type	PZT Number	Capacitance (nF)
Independent Sensor	Single PZT sensor (PZT_3_)	1.60
	Single PZT sensor (PZT_6_)	1.55
	Single PZT sensor (PZT_9_)	1.58
Series connection	Two PZT sensors (PZT_3_, PZT_6_)	0.90
	Three PZT sensors (PZT_3_, PZT_6_, PZT_9_)	0.70
Parallel connection	Two PZT sensors (PZT_3_, PZT_6_)	2.55
	Three PZT sensors (PZT_3_, PZT_6_, PZT_9_)	3.55

**Table 2:** Comparison of CSSN, CPSN, CHSN and NSN.

Network Size	Network Type	Number of Sensors	Number of Channels
3 × 3	NSN	9	9
	CSSN	18	6
	CPSN	18	6
	CHSN	9	5
5 × 5	NSN	25	25
	CSSN	50	10
	CPSN	50	10
	CHSN	25	13
10 × 10	NSN	100	100
	CSSN	200	20
	CPSN	200	20
	CHSN	100	55
*M* × *N*	NSN	*M* × *N*	*M* × *N*
	CSSN	2 × *M* × *N*	*M* + *N*
	CPSN	2 × *M* × *N*	*M* + *N*
	CHSN	*M* × *N*	(*M* × *N* + *N*)/2, *N* is even
			(*M* × *N* − *M* + *N* + 1)/2, *N* is odd

**Table 3:** The impact region localization results on the glass fiber laminate plate.

Impact Energy Level	Network Type	Impact Times	Correct Times	Accuracy Rate
1	CSSN	225	214	95.1%
	CPSN	225	210	93.3%
	CHSN	225	218	96.9%
2	CSSN	225	221	98.2%
	CPSN	225	220	97.8%
	CHSN	225	224	99.6%
3	CSSN	225	222	98.7%
	CPSN	225	217	96.4%
	CHSN	225	224	99.6%

**Table 4:** The number of actual impacts and monitoring results.

Impact Energy Level	Network Type	Impact Times	Correct Times	Accuracy Rate
1	CSSN	625	583	93.3%
	CPSN	625	575	92.0%
	CHSN	625	591	94.6%
2	CSSN	625	589	94.2%
	CPSN	625	582	93.1%
	CHSN	625	595	95.2%
3	CSSN	625	590	94.4%
	CPSN	625	581	93.0%
	CHSN	625	593	94.9%

## Abbreviations

The following abbreviations are used in this manuscript:

ASCS	Aircraft Smart Composite Skin
CDS	Characteristic Digital Sequence
CHSN	Continuous Heterogeneous Sensor Network
CPSN	Continuous Parallel Sensor Network
CSSN	Continuous Series Sensor Network
DR	Duration of the Rise of the characteristic digital sequence
EEPROM	Electrically Erasable Programmable Read Only Memory
EWF	Energy-Weighted Factor
FPGA	Field-Programmable Gate Array
IRM	Impact Region Monitor
NSN	Normal Sensor Network
OFRE	Order of the First Rising Edge of the characteristic digital sequences
PZT	Piezoelectric
SHM	Structural Health Monitoring
UAV	Unmanned Aerial Vehicle
PZT	Piezoelectric

## References

[1] McEvoy M.A., Correll N. (2015). Materials that couple sensing, actuation, computation, and communication. Science.

[2] Guo Y., Li Y.H., Guo Z., Kim K., Chang F.K., Wang S.X. (2016). Bio-inspired stretchable absolute pressure sensor network. Sensors.

[3] Gibson R.F. (2010). A review of recent research on mechanics of multifunctional composite materials and structures. Compos. Struct..

[4] Foote P.D. (2015). Integration of structural health monitoring sensors with aerospace, composite materials and structures. Materialwiss. Werkst..

[5] Yin F., Ye D., Zhu C., Lei Q., Huang Y. (2017). Stretchable, Highly Durable Ternary Nanocomposite Strain Sensor for Structural Health Monitoring of Flexible Aircraft. Sensors.

[6] Soutis C. (2005). Carbon fiber reinforced plastics in aircraft construction. Mater. Sci. Eng. A.

[7] Marsh G. (2010). Airbus A350 XWB update. Reinf. Plast..

[8] Boeing 787 Dreamliner. http://widebodyaircraft.nl/b787.htm.

[9] Staszewski W.J., Mahzan S., Traynor R. (2009). Health monitoring of aerospace composite structures–Active and passive approach. Compos. Sci. Technol..

[10] Yang F.J., Cantwell W.J. (2010). Impact damage initiation in composite materials. Compos. Sci. Technol..

[11] ARP6461 S.A.E. (2013). Guidelines for Implementation of Structural Health Monitoring on Fixed Wing Aircraft.

[12] Park J., Ha S., Chang F.K. (2009). Monitoring impact events using a system-identification method. AIAA J..

[13] Qiu L., Yuan S., Zhang X., Wang Y. (2011). A time reversal focusing based impact imaging method and its evaluation on complex composite structures. Smart Mater. Struct..

[14] Ciampa F., Meo M. (2012). Impact detection in anisotropic materials using a time reversal approach. Struct. Health Monit..

[15] Park B., Sohn H., Olson S.E., DeSimio M.P., Brown K.S., Derriso M.M. (2012). Impact localization in complex structures using laser-based time reversal. Struct. Health Monit..

[16] Chen C., Li Y., Yuan F.G. (2012). Impact source identification in finite isotropic plates using a time-reversal method: Experimental study. Smart Mater. Struct..

[17] Zhong Y., Yuan S., Qiu L. (2015). Multi-impact source localisation on aircraft composite structure using uniform linear PZT sensors array. Struct. Infrastruct. Eng..

[18] Si L., Baier H. (2015). Real-time impact visualization inspection of aerospace composite structures with distributed sensors. Sensors.

[19] Merlo E.M., Bulletti A., Giannelli P., Calzolai M., Capineri L. (2017). A Novel Differential Time-of-Arrival Estimation Technique for Impact Localization on Carbon Fiber Laminate Sheets. Sensors.

[20] Zhu J., Parvasi S.M., Ho S.C.M., Patil D., Ge M., Li H., Song G. (2017). An innovative method for automatic determination of time of arrival for Lamb waves excited by impact events. Smart Mater. Struct..

[21] Huo L., Li X., Chen D., Li H., Song G. (2017). Identification of the impact direction using the beat signals detected by piezoceramic sensors. Smart Mater. Struct..

[22] Ren Y., Qiu L., Yuan S., Su Z. (2017). A diagnostic imaging approach for online characterization of multi-impact in aircraft composite structures based on a scanning spatial-wavenumber filter of guided wave. Mech. Syst. Signal Process..

[23] Qiu L., Yuan S. (2009). On development of a multi-channel PZT array scanning system and it’s evaluating application on UAV wing box. Sens. Actuators A Phys..

[24] Wang Q., Hong M., Su Z. (2015). An in-situ structural health diagnosis technique and its realization via a modularized system. IEEE Trans. Instrum. Meas..

[25] Hardware-Acellent Technologies, Inc.. http://www.acellent.com/en/hardware/.

[26] Mitra M., Gopalakrishnan S. (2016). Guided wave based structural health monitoring: A review. Smart Mater. Struct..

[27] Yuan S., Liu P., Qiu L. (2012). A miniaturized composite impact monitor and its evaluation research. Sens. Actuators A Phys..

[28] Liu P., Yuan S., Qiu L. (2012). Development of a PZT-based wireless digital monitor for composite impact monitoring. Smart Mater. Struct..

[29] Qiu L., Yuan S., Liu P., Qian W. (2013). Design of an all-digital impact monitoring system for large-scale composite structures. IEEE Trans. Instrum. Meas..

[30] Qiu L., Yuan S., Mei H., Qian W. (2013). Digital sequences and a time reversal-based impact region imaging and localization method. Sensors.

[31] Kirikera G.R., Shinde V., Schulz M.J., Ghoshal A., Sundaresan M., Allemang R. (2007). Damage localisation in composite and metallic structures using a structural neural system and simulated acoustic emissions. Mech. Syst. Signal Process..

[32] Kirikera G.R., Shinde V., Schulz M.J., Ghoshal A., Sundaresan M.J., Allemang R.J., Lee J.W. (2008). A structural neural system for real-time health monitoring of composite materials. Struct. Health Monit..

[33] Yuan S., Ren Y., Qiu L., Mei H. (2016). A multi-response-based wireless impact monitoring network for aircraft composite structures. IEEE Trans. Ind. Electron..

[34] Qiu L., Yuan S., Shi X., Huang T. (2012). Design of piezoelectric transducer layer with electromagnetic shielding and high connection reliability. Smart Mater. Struct..

[35] Huang Y., Ding Y., Bian J., Su Y., Zhou J., Duan Y., Zhou Y. (2017). Hyper-stretchable self-powered sensors based on electrohydrodynamically printed, self-similar piezoelectric nano/microfibers. Nano Energy.

[36] Liu M., Zeng Z., Xu H., Liao Y., Zhou L., Zhang Z., Su Z. (2017). Ultra-broadband frequency responsive sensor based on lightweight and flexible carbon nanostructured polymeric nanocomposites. Carbon.

[37] Rajagopalan J., Balasubramaniam K., Krishnamurthy C.V. (2006). A single transmitter multi-receiver (STMR) PZT array for guided ultrasonic wave based structural health monitoring of large isotropic plate structures. Smart Mater. Struct..

[38] Giridhara G., Rathod V.T., Naik S., Mahapatra D.R., Gopalakrishnan S. (2010). Rapid localization of damage using a circular sensor array and Lamb wave based triangulation. Mech. Syst. Signal Process..

[39] Rathod V.T., Mahapatra D.R. (2011). Ultrasonic Lamb wave based monitoring of corrosion type of damage in plate using a circular array of piezoelectric transducers. NDT E Int..

[40] Li F., Peng H., Meng G. (2014). Quantitative damage image construction in plate structures using a circular PZT array and lamb waves. Sens. Actuators A Phys..

[41] Ambroziński Ł., Stepinski T., Uhl T. (2015). Efficient tool for designing 2D phased arrays in lamb waves imaging of isotropic structures. J. Intell. Mater. Syst. Struct..

